# Correlations between RNA and protein expression profiles in 23 human cell lines

**DOI:** 10.1186/1471-2164-10-365

**Published:** 2009-08-07

**Authors:** Marcus Gry, Rebecca Rimini, Sara Strömberg, Anna Asplund, Fredrik Pontén, Mathias Uhlén, Peter Nilsson

**Affiliations:** 1 Department of Proteomics, School of Biotechnology, AlbaNova University Center, KTH – Royal Institute of Technology, S-10691 Stockholm, Sweden; 2 Department of Genetics & Pathology, Rudbeck Laboratory, Uppsala University Hospital, S-75185 Uppsala, Sweden

## Abstract

**Background:**

The Central Dogma of biology holds, in famously simplified terms, that DNA makes RNA makes proteins, but there is considerable uncertainty regarding the general, genome-wide correlation between levels of RNA and corresponding proteins. Therefore, to assess degrees of this correlation we compared the RNA profiles (determined using both cDNA- and oligo-based microarrays) and protein profiles (determined immunohistochemically in tissue microarrays) of 1066 gene products in 23 human cell lines.

**Results:**

A high mean correlation coefficient (0.52) was obtained from the pairwise comparison of RNA levels determined by the two platforms. Significant correlations, with correlation coefficients exceeding 0.445, between protein and RNA levels were also obtained for a third of the specific gene products. However, the correlation coefficients between levels of RNA and protein products of specific genes varied widely, and the mean correlations between the protein and corresponding RNA levels determined using the cDNA- and oligo-based microarrays were 0.25 and 0.20, respectively.

**Conclusion:**

Significant correlations were found in one third of the examined RNA species and corresponding proteins. These results suggest that RNA profiling might provide indirect support to antibodies' specificity, since whenever a evident correlation between the RNA and protein profiles exists, this can sustain that the antibodies used in the immunoassay recognized their cognate antigens.

## Background

The Central Dogma of molecular biology, states that "DNA makes RNA makes proteins" suggesting there is a direct relationship between mRNA and protein levels. This assumed relationship is the basis for numerous transcript-profiling experiments, often based on microarray analysis to identify genes that are up- and down-regulated under normal or disease conditions. The underlying assumption is that differences in mRNA levels are manifested in different phenotypes as a result of differences in protein levels. Accordingly, correlations between the differential expression of specific mRNAs and corresponding proteins have been found in numerous studies [1], many of which have been shown to have clear biological relevance [2,3]. Several studies have also found significant general correlations between RNA levels and protein levels [4-10], usually using data on RNA abundance acquired from platforms such as microarrays and Serial Analysis of Gene Expression (SAGE), in conjunction with data on the abundance of corresponding proteins derived from mass spectrometry (MS) analyses.

The major conclusions drawn from these studies have been that there are significant general correlations between levels of RNA species and corresponding protein products, but also considerable variation in these correlations. For instance, Lu et al found significant correlations between RNA and protein levels of 0.66 and 0.48 in two simple, unicellular organisms (yeast and *Escherichia coli *[8]), but indications that the number of proteins per transcript vary widely. In the cited study MS data and a trained classifier were used to obtain accurate estimates of protein abundance in the complex samples, microarrays were used to determine RNA levels, and products of 346 and 437 genes were used in the yeast and *E. coli *correlation analyses, respectively. Further, in a study published in 1999, Gygi et al investigated 150 genes using SAGE, 2D-gels and MS data, and found a correlation of 0.91 for all analyzed genes, but when a few highly expressed RNA and protein products were excluded the correlation decreased to 0.36 [7].

Similarly, in an analysis of NCI-60 cell lines based on RNA and reverse phase protein arrays, Shankavaram et al found a significant mean correlation between RNA and protein levels, and showed that the correlations were substantially stronger for some gene categories than others [9]. They also found that the distribution of correlation coefficients is bimodal; one group of gene products had a mean correlation of 0.71, while another group had a mean correlation of 0.28. Further, Gene Ontology theme enrichment analysis indicated that the genes with high correlations were mainly involved in the maintenance of cellular processes and structural properties. Greenbaum et al have also shown that gene products associated with certain characteristics, such as high Codon Adaptation Indices (CAI) and/or ribosomal occupancy, seem to have significantly higher correlations with corresponding proteins than the main population of gene products [11].

Thus, interesting data on the degrees of correlation between mRNA and protein levels in various organisms have been acquired, and intriguing variations in this respect between different sets of genes have been detected. However, although MS can provide quantitative data, it has been a bottleneck in analyses of large numbers of gene products. Hence, although several hundred gene products were analyzed in some of the cited studies they still covered small proportions of the total analyzed genomes, so the general conclusions should be treated with some caution. Thus, general patterns of correlation between mRNA and protein levels have not yet been fully established, raising questions about the validity of large-scale comparative mRNA and protein expression profiling, and true, global patterns of relationships between levels of mRNAs and proteins encoded by specific genes still remain to be elucidated. Therefore, in an attempt to compare mRNA and protein levels at a larger scale we have analyzed RNA and protein expression profiles, using cDNA and oligo array data in conjunction with immunohistochemical data, in 23 human cell lines

## Results

### Experimental design

**Figure 1:**
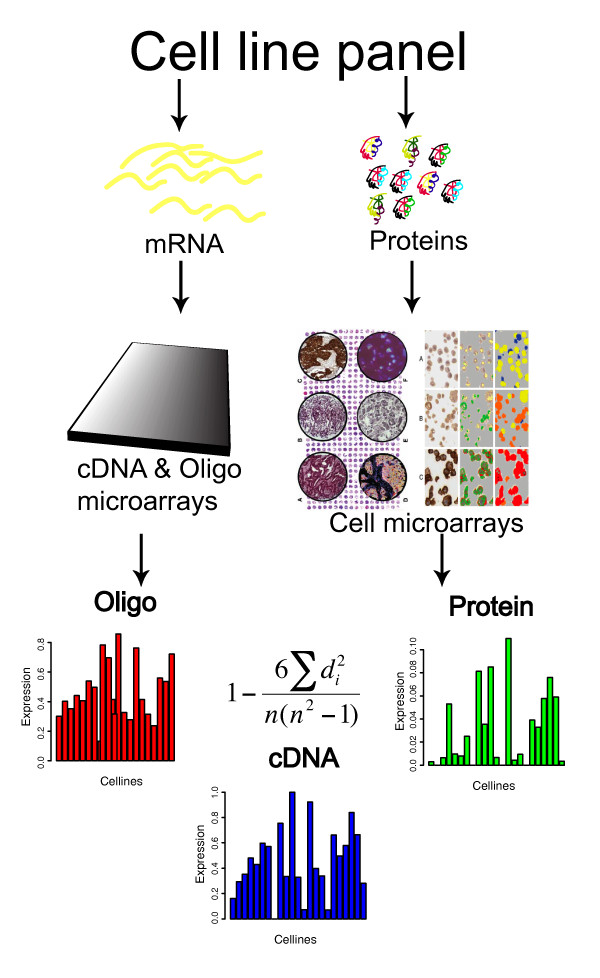
**Outline of the experimental procedure**. RNA expression profiles were generated using cDNA and oligo microarrays, and protein expression levels were generated using immunohistochemical staining of cell microarrays with antibodies from the Human Protein Atlas initiative. The expression levels were measured in each assay in each of the 23 cell lines. For each of the 1066 gene products for which data were obtained from all three platforms, the Spearman correlation coefficients between the RNA _oligo_-protein, RNA _cDNA_-protein and RNA _oligo_-RNA _cDNA _datasets were calculated. The equation of the Spearman correlation calculation is shown in the Figure.

Correlations between levels of RNA and corresponding proteins across 23 cell lines (listed in Additional file 1) were evaluated by comparing immunohistochemical protein expression profiles with transcriptomic data from cDNA and oligo microarrays, as illustrated in Figure 1 and outlined below. The proteomic data used in this large-scale comparison were obtained from 4400 antibody profiles generated in the Human Protein Atlas (HPA) initiative , by applying both antibodies produced in the HPA initiative, and others obtained from various commercial antibody (CAB) vendors, to cell microarrays, and subsequent immunohistochemical staining, following procedures that have been shown to yield data with low intra- and inter-slide variation [12]. In order to further increase the robustness, the immunohistochemical data were all quantified using automated imaging software [13] to scan images of stained glass slides, on each of which cells representing all 23 lines were present. The software quantifies the overall abundance of detected proteins by estimating intensity parameters using a fuzzy algorithm, which provides more robust estimates of quantities of expressed proteins than manual image analysis, since it does not rely on the experience or alertness of the interpreter [14,15]. In order to obtain robust, valid comparative RNA expression values, total RNA was extracted from the same batch of cell lines, converted into Cy5-labeled cDNA, and hybridized in replicates together with a common Cy3-labeled reference, to both cDNA (30 k) and oligo (34 k) spotted microarrays. As detailed in Additional file 1, the cell lines originate from diverse human cancerous tissues, including lung, male and female reproductive system, lymphoid, myeloid, brain, skin and breast tissues.

### Examples of correlation coefficients

**Figure 2:**
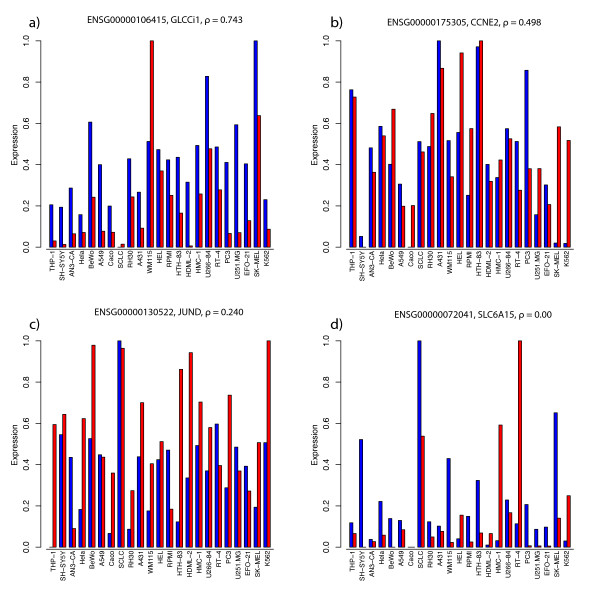
**Four examples of different correlation coefficients: 0.743, 0.498, 0.240 and 0.00 (from the top left to the bottom right) between expression levels of RNA and protein gene products with corresponding Ensembl IDs (ENSG00000106415, ENSG00000175305, ENSG00000130522 and ENSG00000072041, respectively)**. The two lines indicate RNA expression levels measured in oligo microarray (blue) and protein expression analyses (red), across 23 cell lines. The values shown have been adjusted to a comparable scale, by adding the absolute value of the lowest RNA oligo value (which is always negative) to all RNA oligo values. All values (RNA oligo and protein) have then been divided by the highest value for the RNA oligo data. This gives measurements on a comparable (0 – 1) scale and the correlation coefficient remains the same as before the adjustment.

Gene-specific RNA and protein expression profiles were compared, across the 23 cell lines, using Spearman correlation coefficients. To illustrate the comparative profiles utilized in the analysis, examples of profiles with correlation coefficients ranging between 0 – 0.75 are shown in Figure 2. It should be noted that the assays used (see *Methods *for details) provide indications of relative rather than quantitative levels of expression, but since expression profiles across multiple cell lines were examined, the correlation coefficients can still be meaningfully compared. Spearman's correlation coefficients were used since some of the data compared in this study are linear but others are logarithmic, so rank-based coefficients yield more robust estimates of correlation than linear coefficients, such as Pearson's coefficients, which could be strongly biased by extreme values. Further, in cases where there is high variance, as illustrated in Figure 2d, the probability of stochastic phenomena having similar effects on the relative strength of expression of both transcripts and corresponding proteins in assays with each of the 23 cell lines is very low, so significant correlation coefficients are unlikely to be obtained, using a rank-based approach, if the high variability is due largely to stochastic effects. Examples of profiles with intermediate correlation coefficients are shown in Figure 2b and 2c, to illustrate the influence of variations in the strength of expression of transcripts relative to that of corresponding proteins across the cell lines.

### Selection of the analyzed genes

After strict quality filtering of the three expression datasets (see *Materials and methods *for details), data on levels of 1066 gene products with unique Ensembl gene IDs provided by all three platforms remained. The effects of applying different filtering criteria are illustrated by the oligo array data shown in Additional file 2, which indicates that the stringency of the filtration (in terms of permitted numbers of missing data points for specific genes and cell lines included in the analysis) has minor effects on the mean correlation coefficient. However, the highest mean correlation coefficients were obtained in all three comparisons when no missing values were accepted, so only profiles for which data from all cell lines were available from each of the platforms were used. Other tested options were to include average expression values for gene products that had more than one counterpart in the data yielded from another platform across all the cell lines, or the best matched pairs, based on either sequence similarities or correlation coefficients. However, the distributions of correlation coefficients obtained with these approaches did not significantly differ from those yielded by averaging the expression values obtained with multiple probes.

High proportions of the 1066 gene products for which data were available in all three of the filtered datasets were detected by a single probe or antibody (75%, 55% and 39% in the Protein, Oligo and cDNA datasets, respectively). Two or more representatives of the remaining gene products were detected, i.e. replicates resulting in multiple data points (Additional file 3).

### Correlations between RNA and protein levels

**Figure 3:**
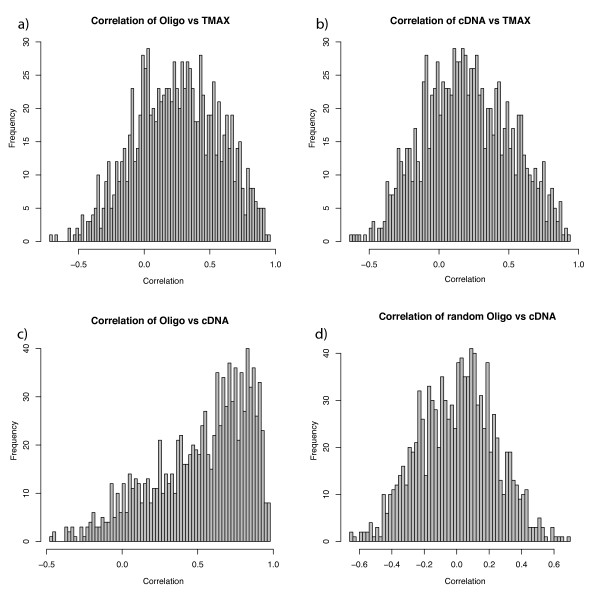
**Histograms of all correlation coefficients for each gene product obtained from each of the three comparisons, and one showing those of randomly picked Ensembl ID pairs**. Figures 3a – 3c show the RNA _oligo _versus protein profiles, RNA _cDNA _versus protein profiles, and the RNA _oligo _versus RNA _cDNA _profiles, which yielded mean correlation coefficients of 0.25, 0.20 and 0.52, respectively. The distributions of correlation coefficients between RNA values obtained using both RNA platforms and the protein values have Gaussian shapes, but with some bimodal characteristics, in which most of the data points are centered at the respective mean, but shoulders can be seen at correlation coefficients of ~0.5 – 0.7. For the RNA assay correlations the distributions follow a beta distribution. The randomly picked pairs have a mean value close to zero, indicating that there is no apparent bias in the data set.

In order to calculate pairwise correlation coefficients between gene product pairs across all cell lines, three matrices of 1066 gene product pairs and 23 cell lines were constructed using data on all of the gene products that were quantified by all three platforms. The mean Spearman correlation coefficients for the 1066 comparisons in the oligo microarray versus protein, cDNA microarray versus protein and oligo microarray versus cDNA microarray profile comparisons were 0.25, 0.20 and 0.52, respectively, while the corresponding median values were 0.26, 0.19 and 0.60, respectively. Histograms of the Spearman correlation coefficient distributions are displayed in Figure 3.

### Genes with correlated RNA and protein expression levels

**Figure 4:**
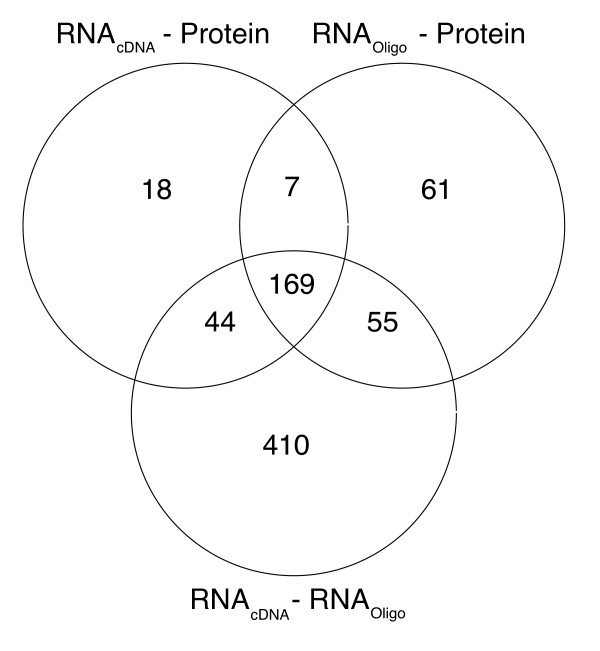
**Venn diagram showing numbers of highly correlating gene products identified in the comparison of data obtained from each permutation of platforms**. The gene products that had correlations 0.445 in each comparison (RNA_oligo_-protein, RNA_cDNA_-protein and RNA_oligo_-RNA_cDNA_) were compared with those identified in the other assays. 169 gene products with such correlations were identified in all three comparisons, equivalent to 63% and 82.5% of those found in the RNA _oligo_-protein and RNA_cDNA_-protein comparisons, respectively. The numbers of gene products with correlation coefficients 0.445 in the RNA _oligo_-protein, RNA_cDNA_-protein and RNA_oligo_-RNA _cDNA _comparisons were 292, 238 and 678, respectively.

To identify transcripts and corresponding proteins with significantly correlated expression profiles a correlation coefficient cutoff of 0.455 was applied, based on the null hypothesis that the mean correlation between given RNA species and proteins with different Ensembl IDs is 0, and applying a t-score threshold of 2.08 (corresponding to the 95% confidence interval). To validate this assumption the mean correlation coefficient was calculated for 1000 randomly selected Ensembl ID pairs, and found to be -0.001, indicating that the Null assumption is valid. Further, since multiple tests were applied, Benjamini-Hochberg multiple testing adjustment was used. Hence, in subsequent analyses a cut-off level of 0.445 was applied. The number of gene product pairs for which correlations 0.455 were found in the Oligo-Protein, cDNA-Protein and Oligo-cDNA comparisons were 292, 238 and 678, respectively. The Ensembl gene IDs corresponding to gene product pairs with correlations exceeding 0.455 in each of these comparisons were then used to construct a Venn diagram illustrating the numbers shared in each comparison (Figure 4). The 169 genes (16%) meeting the criteria described above in the datasets generated by all three platforms are tabulated in Additional file 4. The proportions of products detected by commercial antibodies (CAB) and Human Protein Atlas antibodies (HPA) among the 169 Ensembl IDs were the same as those used to generate the initial dataset (38% CAB, 62% HPA). The numbers of Ensembl gene IDs in the oligo microarray versus cDNA microarray comparison and gene products in the comparisons of either of the RNA and protein comparisons yielding correlations > 0.455 were 678 (64%) and 354 (33%), respectively. Hence, a third of the antibodies could be validated based on a stringent comparison of correlations between the RNA and protein levels across all cell lines.

### Gene ontology analysis

Analysis of the cellular compartment and biological process Gene Ontology (GO) themes [16] of the gene products with correlation coefficients >0.445 identified in the three RNA-RNA and RNA-protein comparisons described above yielded varying results, and only a few significantly enriched GO themes were detected (after adjustment for multiple testing). It should be noted that since the dataset is rather small for such hypergeometric statistical tests the results are highly sensitive to relatively minor variations. However, among the 169 common Ensembl gene IDs, significant enrichment was found of genes associated with the cytoskeleton and adherent junctions in the cellular compartment ontology analysis, and of genes associated with cellular motility and other maintenance-related categories in the biological function ontology analysis (Additional file 5 and 6).

To assess the possibility that the correlation coefficients could be dependent on the RNA array signal intensity, linear regression was applied to the mean signal intensities and correlation coefficients. Using all 1066 gene product pairs a positive, but weak relationship was found (*m = *0.034, *p *= 4.39e-05) in values obtained from the RNA oligo assay, indicating that an increase in signal intensity slightly increased the correlation. The corresponding relationship for the results from the cDNA assay was extremely weak (*m = *0.15e-05, *p *= 0.98), indicating that variations in the signal intensity had virtually no effect on correlations of the data provided by the cDNA arrays. Hence, the oligo microarray analysis yielded larger differences in correlations obtained with high and low intensity probes than the cDNA analysis.

### Global expression profiling

**Figure 5:**
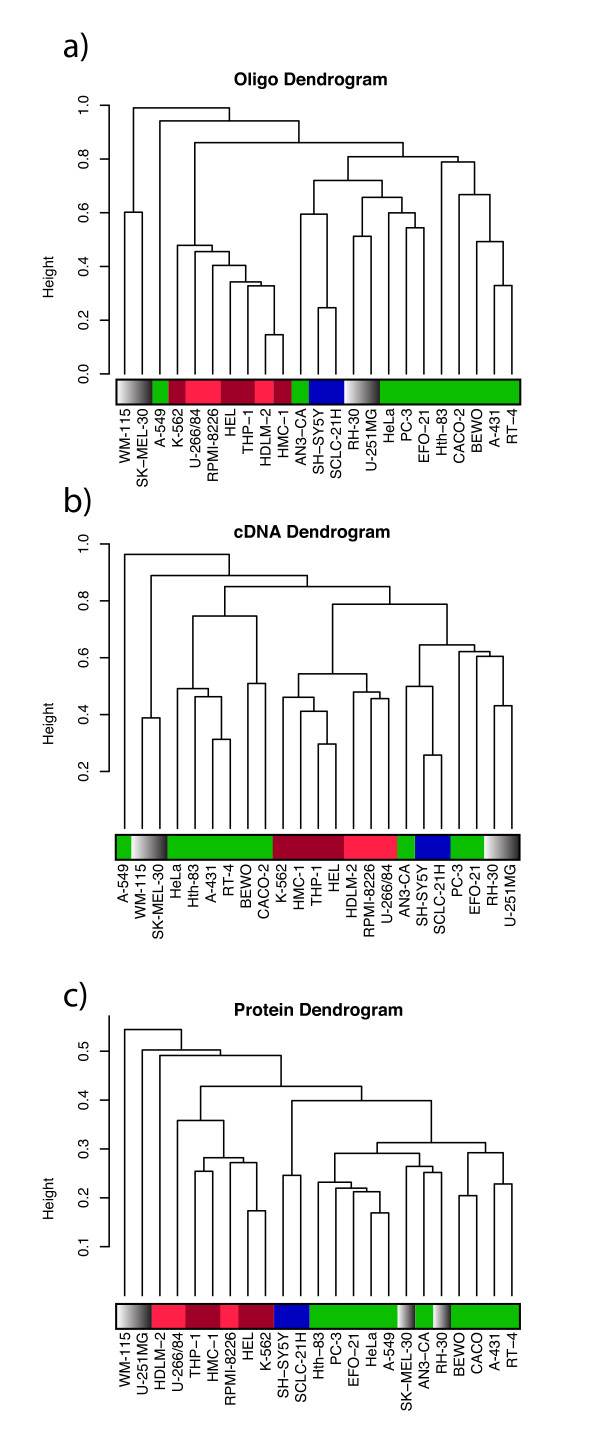
**Dendrograms of hierarchical clusterings based on 1 – Pearson correlation coefficient metrics**. The expression levels of 169 Ensembl gene IDs with correlation coefficients >0.445 for which data were available in all three comparisons across 23 cell lines measured in each assay were utilized to cluster the data into three individual clusters. The cell lines are colored depending on their origins; red, deep-red, grey, green and blue indicate cells of: lymphoid; myeloid; melanoma, glioma and sarcoma; carcinoma and neuronal origins, respectively.

To investigate the relationships of global expression profiles in the examined human cell lines, dendrograms were generated for each of the RNA assay and protein datasets, based on the similarity of the expression levels of the 169 gene products that were common to all comparisons shown in the Venn diagram. The dendrograms, colored according to their tissue of origin in Figure 5, indicate that there were both similarities and variations in the expression patterns detected by the three assays. The two dendrograms based on RNA data (Figure 5a and 5b) have high similarity (cophenetic correlation = 0.84), but different sub-clustering patterns compared with the protein data. Another notable feature of the clusters is that the adherent cells and the suspension-growing cells (all of which have hematological origins, except the SCLC cell line) are divided into different sub-clusters. The RNA-based assays seem to separate the cell lines better than the protein assays. The cophenetic correlations between the oligo RNA and protein datasets, and the cDNA and protein datasets, are 0.32 and 0.22, respectively; in the same range as the mean correlation coefficients. Dendrograms created using the full data set of 1066 Ensembl IDs have greater similarity (Additional file 7); the cophenetic correlation coefficients for the cDNA versus Oligo, Oligo versus Protein, cDNA versus Protein assays being 0.78, 0.45 and 0.32, respectively. The cophenetic correlation coefficients between the dendrograms generated using the subset of 169 Ensembl IDs and the larger dataset with 1066 Ensembl IDs are 0.64, 0.71 and 0.78 for the cDNA, Oligo and Protein datasets, respectively.

### RNA assay validation using real time reverse transcription polymerase chain reactions

In order to validate the results from the two RNA microarray assays, Real time reverse transcription polymerase chain reaction (RT-PCR) analysis was applied, in which products of 14 genes were measured in duplicates across eight cell lines. The correlations obtained for all 14 genes in a subsequent RT-PCR-oligo RNA array comparison (which ranged from -0.26 to 1) and for 10 genes in an RT-PCR-cDNA comparison (which ranged from -0.6 to 0.97) are shown in Additional file 8. In addition, the correlation between 7 different genes and the corresponding protein levels was calculated (Additional file 8), where the mean correlation was 0.58. Further, for four genes per RNA assay linear regression was applied to the correlation coefficients of the RNA array versus RT-PCR results, and the RNA array versus protein data. The slope obtained from the regression analysis was significantly positive (p-value = 0.01), indicating that there was a significant relationship between these correlations (Additional file 9).

## Discussion

To assess the fundamentally important correlation between levels of RNA species and corresponding proteins accurately, reliable estimates of their abundance are clearly required. Equally clearly, quantitative methods that yield highly accurate, absolute estimates of their levels would ideally be applied, and currently the method of choice for quantitative proteomics is mass spectrometry, following several purification and separation steps. This approach can provide high levels of accuracy, sensitivity and specificity, but as yet it is not suitable for large-scale analyses. Alternatively, as in this study, relative levels of proteins across samples of multiple cell lines in tissue microarrays can be determined immunohistochemically, minimizing inter-experimental variation by simultaneously staining samples of all of the lines by each antibody. In addition, various types of microarrays have been developed recently that are capable of providing reliable estimates, in conjunction with various statistical models, of absolute quantities of specific mRNA species in samples from spot intensities [17].

Thus, use of relative techniques like two-color microarray and immunohistochemistry allows levels of large numbers of gene products to be compared in multiple samples. However, it should be recognized that cell lines are model systems that differ in various respects from cells in the organisms from which they are derived, notably many of the regulatory pathways are not present and the chromosomal arrangements are beyond the normal patterns in healthy tissues [18]. So, findings regarding correlations between RNA and corresponding protein levels in them should be interpreted with some caution. Furthermore, since the abundance of RNA and protein is analyzed in samples of cell lines containing several cells, the values used in subsequent correlation analysis are based on averages for the cell line populations, which may be in varying stages of the cell cycle.

Bearing in mind the above provisos, the distributions of the correlation coefficients obtained in both the cDNA and oligo microarray data comparisons with the protein dataset are approximately normal distributed, although when investigating the density function of the distribution there is a tendency towards a minor peak around a mean value of 0.65–0.75, implying that the gene products can may be divided into two major groups that have different degrees of correlation. Further, the minor peak is enhanced when the correlations are based on Pearson correlation coefficients. Shankavaram et al noticed a similar pattern in their study of NCI-60 mammalian cell lines [9]. In contrast, the distribution of cDNA versus oligo microarray correlations had more of a beta shape, indicating that data generated from many pairs of corresponding probes in the two array systems strongly correlated, but some pairs yielded results that correlated poorly, which decreased the mean correlation coefficient. This may have been due to poor sequence overlap, i.e. the probes yielding poor correlations may have hybridized to different parts of transcripts that mapped to the same genes according to data in the Ensembl gene database. The degree of correlation between the cDNA and oligo microarray datasets is consistent with the degrees found in previous analyses [19], but further evaluation of variations between the results of this and previous studies in this respect is beyond the scope of this article. The oligo microarray assay yielded higher correlation coefficients with the protein data than the cDNA microarray assay, probably because the oligo probes had higher specificity, in accordance with expectations due to the lower degree of cross hybridization that generally occurs when shorter probes are used.

The major and minor peaks in the in the histograms of the correlation coefficients between the oligo microarray and protein profiles may correspond to two groups of genes that are regulated by different mechanisms. The genes with high correlations may be regulated solely, or almost solely, at the transcriptional level, in accordance with evidence from the ontological analysis that high proportions of these genes are involved in cellular processes and maintenance, for which there is likely to be little need for complex regulation. In contrast, the weak correlations of the other sets of genes may be due to the effects of complex regulatory mechanisms and/or noise generated in the assays masking subtle changes in mRNA transcripts and protein levels, thereby weakening the correlations.

The weak correlations for gene products with correlation coefficients lower than 0.445 probably have several causes, including various post-transcriptional processes that complicate attempts to obtain accurate estimates of quantities of corresponding mRNAs across the cell lines that are destined for translation. For instance, some mRNAs are strongly retained in the nucleus, which may lead to their levels being over-estimated relative to protein levels. Technical noise generated by the respective platforms (notably due to cross-hybridization in the DNA microarray analyses and variations in the affinity and specificity of the antibodies used in the immunoassays) may also weaken the correlations, and thus increase the proportions of genes with correlation coefficients lower than 0.445. The reason that no correlation was found for certain genes is probably related to the complexity of their regulatory mechanisms, which may weaken their correlations to levels that are not detectable with current techniques, while genes with strong correlations may be regulated solely at the transcriptional level.

The concordance between estimates of RNA levels obtained from the array analyses and the RT-PCR analyses was found to be positively correlated to the correlations found between the RNA and protein levels, but the quantities of transcripts estimated by the RT-PCR assay was not similarly related to the RNA-protein correlations. The number of samples is too small to draw definitive conclusions, but these results suggest that if the accuracy of RNA estimations is increased (based on the correlation with the RT-PCR assay), the correlation between RNA and protein levels is more likely to be high (Additional file 9). In addition, the analysis of RNA levels estimated by RT-PCR showed that the mean correlation is higher than the array based platforms, albeit the number of samples in the analysis is also small. This implies that a more accurate estimate of the RNA levels is likely to increase the overall correlation, and that the cause of low correlation are mainly caused by variable accuracy on the RNA level and not the protein estimates.

We have shown here that the correlation coefficients between RNA and protein profiles for 1066 gene products across 23 cell lines vary widely. The mean correlation coefficient is ~0.3, but the groups of genes represented by a major peak at mean value ~0.3 and a minor peak at mean value 0.65–0.75 have significantly different mean values, which may reflect differences in their regulatory mechanisms. Utilizing RNA data from two independent microarray formats, and immunohistochemical data obtained using antibodies applied in the Human Protein Atlas initiative, we found significant correlations between the RNA and protein profiles of 33% of the gene products. Although transcriptional profiling cannot be considered a high-throughput approach for the validation of affinity reagents, when correlation measurements between RNA and protein levels are available they provide additional information regarding the performance of employed antibodies. Further, when the RNA estimates are highly accurate the correlation between RNA and protein levels has a tendency to increase. However, while high correlation values might support antibody specificities, observed discrepancies between RNA and protein levels do not necessarily imply that the antibodies perform poorly, since they could be due to various biological factors, such as complex gene regulatory mechanisms.

## Methods

### RNA data

The data on gene expression at the RNA level were acquired using microarray technology, as follows. RNA from each of the 23 cell lines was hybridized to internally produced oligo arrays and cDNA microarrays, spotted onto UltraGAPS slides (Corning). The oligos were the human 3.0 set from Operon (Array Express: A-MEXP-706), containing ~37000 probes, representing ~24600 unique genes, while the cDNA microarrays contained ~30000 probes representing ~11800 unique genes (Array Express: A-MEXP-250). The cell lines were hybridized on duplicate (oligo) and duplicate/triplicate (cDNA) arrays. Each cell line was hybridized with Stratagene universal reference RNA, and for each sample 20 μg of RNA was primed with 5 μg random hexamers (Invitrogen). The volume of each sample was adjusted to 18.4 μl using DEPC-treated water. The RNA was denatured at 70°C for 10 minutes, and then renatured on ice for 5 minutes. Reverse-transcription reaction mixture (Invitrogen) and 400 units of Superscript III RT-polymerase were added to yield a final volume of 30 μl containing 1× first-strand buffer (Invitrogen), 0.01 mM DDT (Invitrogen) and 0.5 mM dNTPs (Sigma-Aldrich). The ratio of aminoallyl-modified dUTP to dTTP was 4:1 in the dNTP mixture. The samples were incubated at 25°C for 10 minutes followed by 46°C for 2 hours. The cDNA synthesis was halted by adding 3 μl 0.2 M EDTA (pH 8.0).

Template RNA was removed by adding 4.5 μl 1 M NaOH. The samples were incubated at 70°C for 15 minutes, and then chilled to room temperature, neutralized with 4.5 μl 1 M HCl and purified using the MinElute Reaction Cleanup system (Qiagen), following the manufacturer's recommendations, except that the wash and elution buffers provided with the system were replaced by 80% ethanol and 100 mM NaHCO_3 _(pH 9.0), respectively. The elution step from the column was repeated, generating an eluate of 20 μl. This was mixed with a tenth of the contents of a monofunctional NHS-ester Cy3 or Cy5 dye tube (GE Healthcare), which had been dissolved in DMSO and subsequently dried in a vacuum centrifuge. After 30 minutes incubation in darkness at room temperature, the samples to be hybridized were purified using MinElute columns as instructed by the manufacturer.

### Hybridization of samples

The microarray slides were pre-hybridized for 30 minutes at 42°C in a pre-hybridization solution consisting of 5× SSC, 0.1% SDS (Sigma-Aldrich) and 1% BSA (Sigma-Aldrich) to avoid unspecific hybridization to the glass surface. The slides were subsequently washed in water and isopropanol (Sigma-Aldrich), then dried using a slide centrifuge. The labelled (Cy5) and reference (Cy3) samples were pooled and denatured (3 minutes at 95°C) in a hybridization mixture containing 25% formamide (Sigma-Aldrich), 5× SSC and 0.1% SDS. The mixture was introduced under a lifter-slip cover slip (Erie Scientific) placed on top of the printed array and hybridized for 18–24 hours at 42°C in a water bath. Following hybridization the slides were washed with increasing stringency using 2× SSC and 0.1% SDS at 42°C, followed by 0.1× SSC and 0.1% SDS at room temperature and finally by five repeated washes with 0.1× SSC at room temperature.

Following hybridization the arrays were scanned at 10 μm resolution using an Agilent G2565BA scanner (Agilent Technologies, Santa Clara, CA, USA), with the photomultiplier set to 100% for each laser. The acquired images were analyzed using the irregular gridding algorithm in GenePix Pro 5.1 (Molecular Devices), and the resulting data were imported into the R environment for statistical processing and visualization [20]. The intensities were extracted from the median foreground intensity in the 532 nm and 635 nm channels. The features were filtered based on the data from GenePix and manual inspection of the slides, by removing spots that were either not found by the image software or were marked as bad spots due to the presence of dust particles or contact with adjacent spots on the array.

The intensities of signals from features within each array were normalized by print-tip Lowess normalization [21,22]. The log2 values of the ratios of the two normalized intensities (abbreviated M value, 1/2(log_2_(F635/F532)) and the product of the intensities (abbreviated A value, 1/2(log_2_(F532 * F635))) for all features were then calculated. The intensities were also normalized across arrays using a median absolute deviation scaling method [22].

The DNA microarray data have been submitted to Array Express.

### Cell microarray production

The cell samples were assembled in a cell microarray (CMA) as previously described by Andersson et al. [12]. Briefly, cells were fixed in formalin and dispersed in agarose. The generated cell pellets were then histoprocessed and embedded in paraffin, resulting in donor blocks for CMA production. From each cell donor block duplicate 0.6 mm punches were taken and placed in one recipient CMA.

### Immunohistochemistry and image analysis

As previously described by Stromberg et al., antibodies generated in the Human Protein Atlas project were used for immunohistochemical staining of CMA sections [13]. All stained CMA sections were scanned using a Scanscope T2 automated slide-scanning system (Aperio Technology) and generated TIFF images representing separated cell spots were analyzed using TMAx automated image analysis software (Beecher Instruments). The software automatically identifies cells and detects immunostaining, generating an output file containing information about staining intensity, fractions of positive cells, numbers of cells present per spot etc.

### Protein quantification

Protein quantification scores were calculated using TMAx output parameters of staining intensity per unit area and the number of cells present in each cell spot. Spots with insufficient cells (<20) were excluded from further analysis. Since the staining intensity reflects the amount of protein present in a cell, signals from areas in each cell with weak, moderate and strong staining were summed, weighting moderate and strong signals with arbitrary coefficients of 2 and 3, respectively. The parameters used for determining the cut-off levels for each staining category were jointly determined by experienced pathologists and the software developer. The summed values were then divided by the number of cells present in the respective spots, generating average values of protein expression level per cell. In order to correct for bias introduced by the correlation between cell size and the level of protein expression, as described by Lundberg et al. [23], the protein expression levels obtained per cell were adjusted with respect to cell size. Using image analysis data, the average cross-sectional area for each cell line was calculated from 100 CMAs, and by setting the cell size of the largest cell to 1, a relative average size for each cell type was computed. Finally, values of protein expression level were divided by the relative average cell sizes, yielding apparent protein concentration values.

### Numerical analysis

In this study, datasets from two RNA platforms (oligo and cDNA microarrays), and one protein (immunohistochemical) dataset were obtained. To enable RNA and protein levels to be compared the gene products must have corresponding identities. Therefore, matrices containing the intensity data for corresponding gene products, based on shared Ensembl Gene IDs, were compiled. In some cases the overlap of the IDs was not 1:1, but instead one Ensembl gene ID identified in the data obtained from one platform had multiple counterparts in the data obtained from another platform. In such cases values for the multiple hits were averaged. In addition, an analysis was performed in which only the most strongly correlating Ensembl pairs, from a collection of pairs with multiple, matching Ensembl IDs, were utilized. This analysis did not yield any significant differences in overall mean correlation coefficients compared to the method in which averages were used. For the respective platform, the intensity values were measured, more specifically, log 2 ratios (M-values) of the intensity values from the cDNA and oligo microarray datasets, and the intensities quantified by the TMAx software for the immunohistochemical data, were used in the correlation analysis

### Data filtration

All datasets were filtered based on Non Available values (NAs), in which each Ensembl gene ID had to have representative values for each cell line, or else it was discarded in the subsequent analysis. The effect of the filtering is illustrated in Additional file 2.

Hence, three matrices were constructed with data for 1066 Ensembl gene IDs in 23 cell lines that were present in the datasets obtained from all three platforms. The Spearman's Rho correlation was then calculated for each Ensembl gene ID pair between the RNA_cDNA_-RNA_oligo_, RNA_cDNA _-protein and RNA_cDNA _-protein datasets.

### Bootstrapping

To investigate whether there were artifacts in the data, or if the different cell lines differed in overall expression levels, randomly sampled gene products were picked to check their correlation coefficients. The mean correlation coefficient was zero, indicating that the dataset was robust.

### Hierarchical clustering

Using the three datasets, a subset of 169 gene products for which data were available from all three platforms were chosen that had higher correlation coefficients than 0.5 in all comparisons to construct three dendrograms, by applying a 1 – Spearman correlation metric and a top-down hierarchical method with average agglomeration.

### RT-PCR

Expression levels for 14 mRNA gene products were analyzed in eight selected cell lines by quantitative real-time PCR using a BioRad iCycler (BioRad Laboratories, Hercules, CA, USA) and SYBR Green-labeling of amplicons. Pairs of genes were analyzed simultaneously, and for each gene a nontemplate control was added. For each run, a general set-up was used consisting of three independent dilution series of a gene-specific plasmid template with known copy number to construct a standard curve as well as triplicates of cell line cDNA templates for quantitative analysis. The standard curve was generated using iCycler software (Optical System Software Version 3.0a), in which the obtained threshold cycles values (*C*t) were plotted against the logarithmic copy numbers of the plasmid dilution series. The *C*t values of the cell lines were fitted to this plot and thus the copy numbers were determined.

The specificity of the priming and amplification was verified with a melt curve for every amplicon. The quantitative real-time PCR was performed in duplicates, and copy number results were averaged, resulting in eight mean copy numbers for each cell line.

## Authors' contributions

MG and RR performed and analyzed all RNA-based experiments. SS and AA performed all protein-based experiments. AA and FP designed and analyzed the protein data. MG performed all statistical analysis. MG, MU and PN conceived and designed the study. PN coordinated the project. All authors contributed and approved the manuscript.

## Supplementary Material

Additional file 1 **Cell line summary**. Description of the cell lines used in the experiments. Click here for file

Additional file 2 **Effect of missing values on the mean correlation coefficient**. Effects on the mean correlation coefficient of applying different filtration criteria, i.e. the number of allowed missing values (0 – 23) in the RNA oligo assay data. The x-axis indicates the number of missing values and the y-axis the mean correlation value. The numbers in the plot indicate the number of remaining data points. The mean correlations, post-filtration, range from 0.214 to 0.237. Click here for file

Additional file 3 **Number of replicate probes and antibodies**. Histograms of gene product probes/antibodies replicates used to measure expression levels of the Ensembl gene ID products identified by each platform. The values are averaged across the cell lines whenever replicate hits are found. Click here for file

Additional file 4 **Summary of correlation coefficients**. Details of the 169 Ensembl gene IDs for which strong correlations were found in comparisons of data obtained from all three platforms, and the correlation coefficients obtained. Click here for file

Additional file 5 **Summary of the biological processes ontology results**. Table showing results from the Biological Process gene enrichment analysis. Each category (Gene ontology term), and the total number of members from the dataset within each category are listed. The expected number within each category, the numbers of members derived from the dataset, and the associated p values (both unadjusted and adjusted using a false discovery rate multiple adjustment method) are also presented. The category name column contains keywords for the respective categories. Click here for file

Additional file 6 **Summary of the cellular compartment ontology results**. Table showing results from the Cellular Compartment gene enrichment analysis. Each category (Gene ontology term), and the total number of members from the data set within each category are listed. The expected number within each category, the numbers of members derived from the dataset, and the associated p values (both unadjusted and adjusted using a false discovery rate multiple adjustment method) are also presented. The category name column contains keywords for the respective categories. Click here for file

Additional file 7 **Dendrograms based on hierarchical clustering of the complete dataset**. Dendrograms from hierarchical clustering based on 1066 genes, using the same clustering procedure as for the smaller subset of 167 Ensembl gene IDs. Click here for file

Additional file 8 **Summary of the array versus RT-PCR results**. (A) Correlations between Oligo array and RT-PCR data (middle column) and the array and protein data (right column). (B) Correlations between cDNA array and RT-PCR data (middle column) and the array and protein data (right column. (C) Correlation between RT-PCR data and the immunoassay data. A "-" sign correspond to a missing value. Click here for file

Additional file 9 **Linear relationship between correlation coefficients from array and RT-PCR intensity signals**. Linear regression of the correlations between the oligo microarray data and the cDNA microarray data (y-axis) and the correlations between each of the array data assays and the RT-PCR assay data (x-axis). The slope is significantly positive (p-value 0.01). Click here for file
