# Supplementary figures and images for: Correlations between RNA and protein expression profiles in 23 human cell lines
Source: BMC Genomics. 2009 Aug 7;10:365. doi: 10.1186/1471-2164-10-365 (PMC2728742; doi:10.1186/1471-2164-10-365)

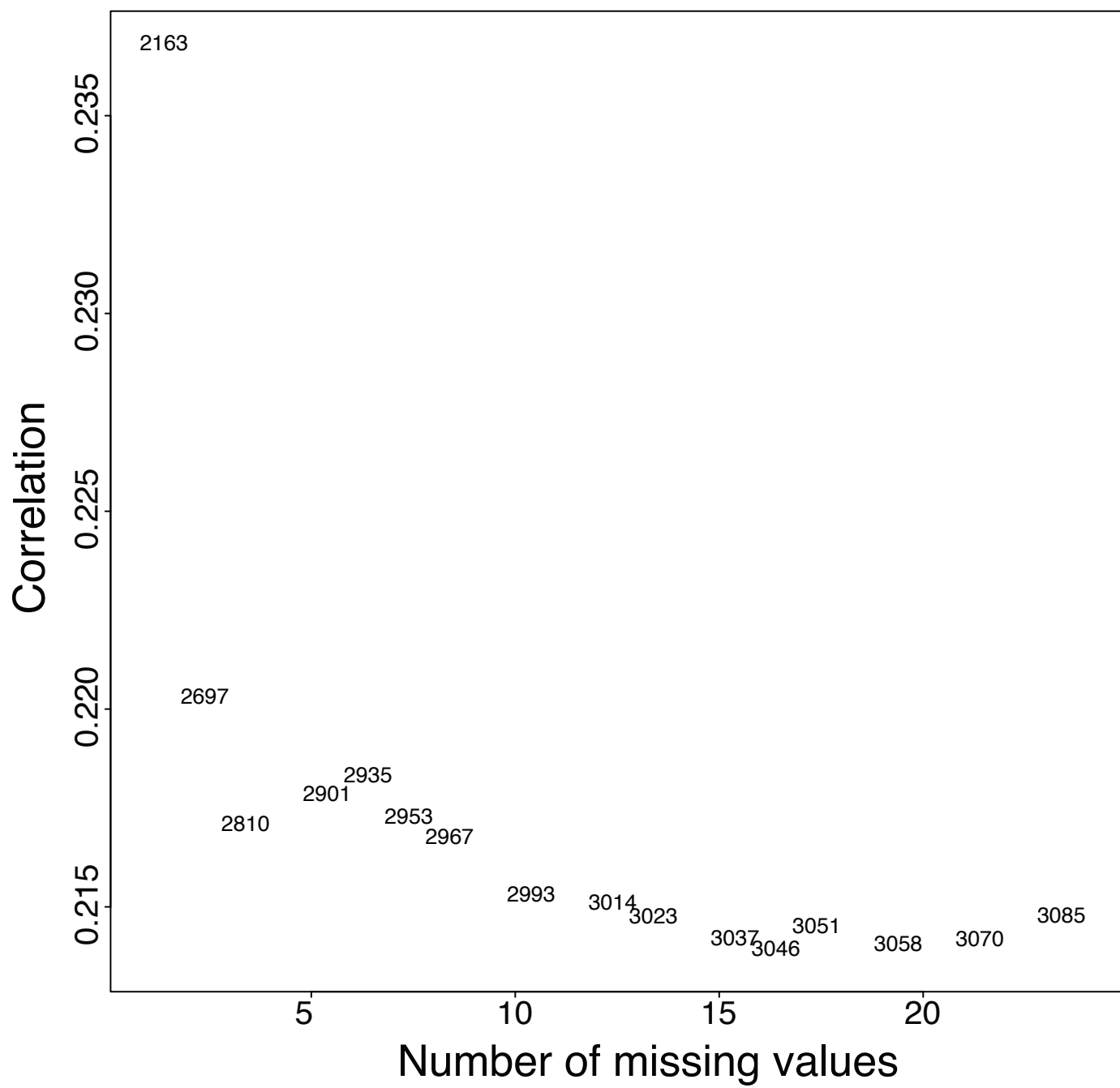

Supplement: Additional file 2 — Effect of missing values on the mean correlation coefficient. Effects on the mean correlation coefficient of applying different filtration criteria, i.e. the number of allowed missing values (0 – 23) in the RNA oligo assay data. The x-axis indicates the number of missing values and the y-axis the mean correlation value. The numbers in the plot indicate the number of remaining data points. The mean correlations, post-filtration, range from 0.214 to 0.237. [file 1471-2164-10-365-S2.pdf]

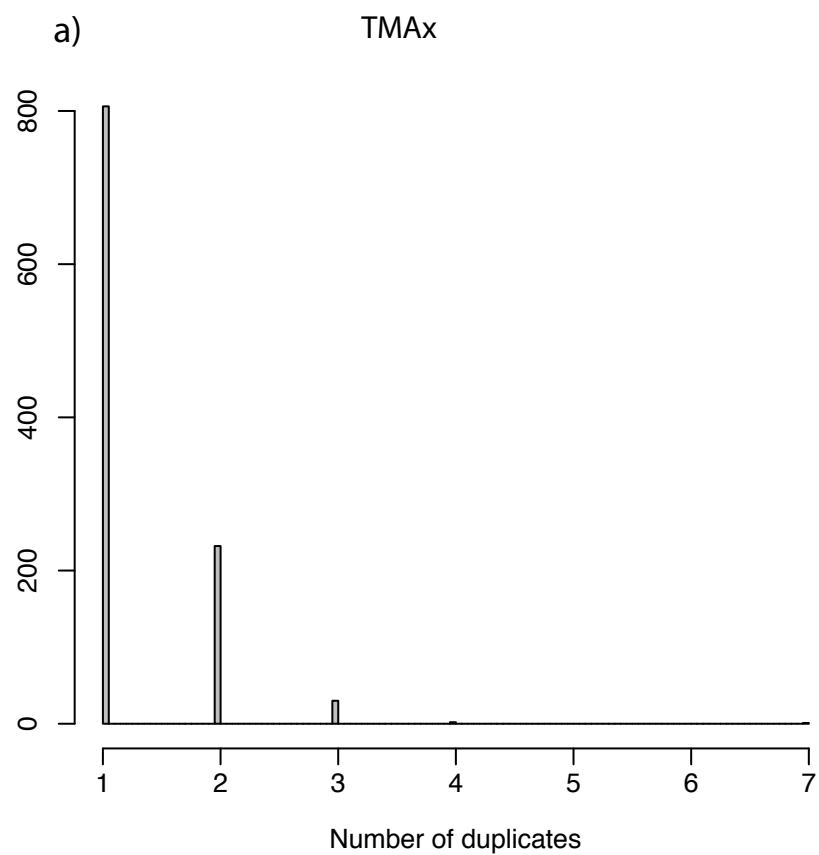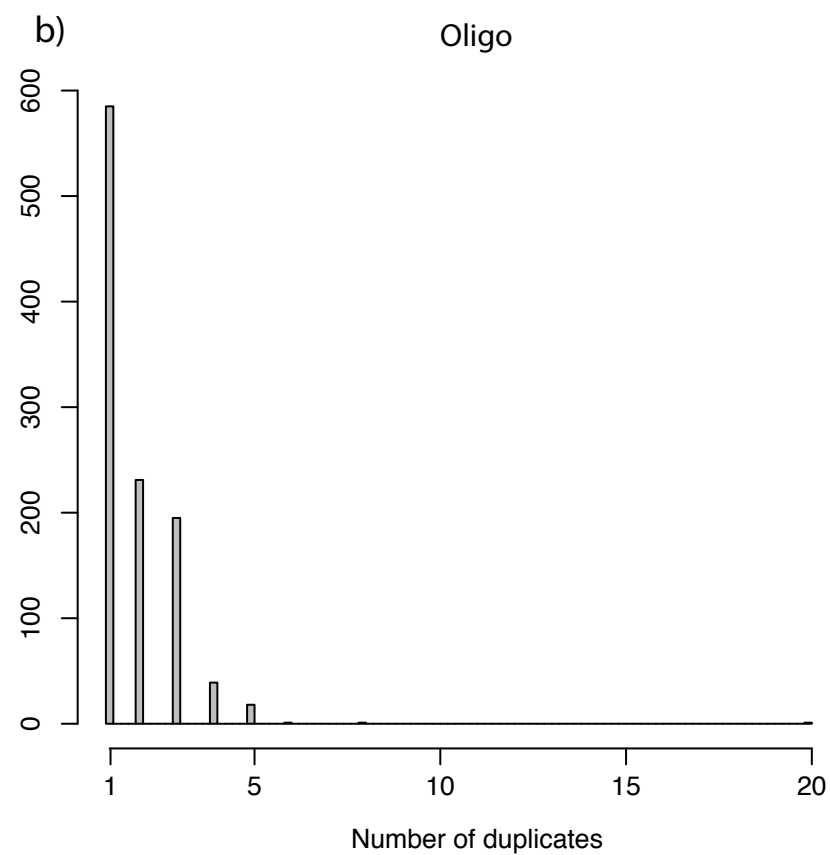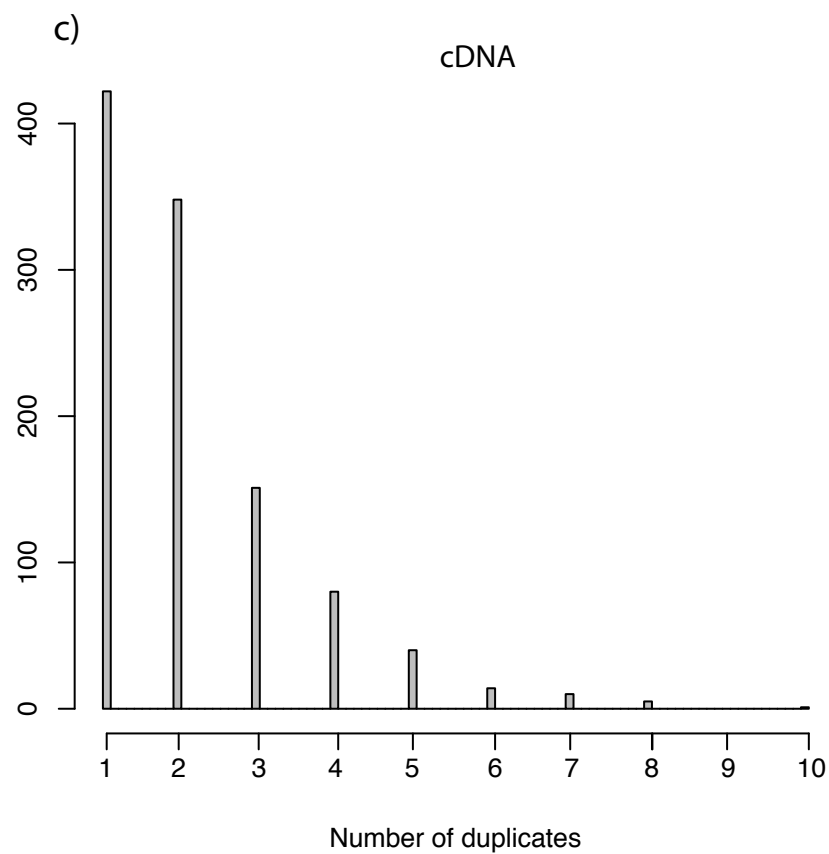

Supplement: Additional file 3 — Number of replicate probes and antibodies. Histograms of gene product probes/antibodies replicates used to measure expression levels of the Ensembl gene ID products identified by each platform. The values are averaged across the cell lines whenever replicate hits are found. [file 1471-2164-10-365-S3.pdf]

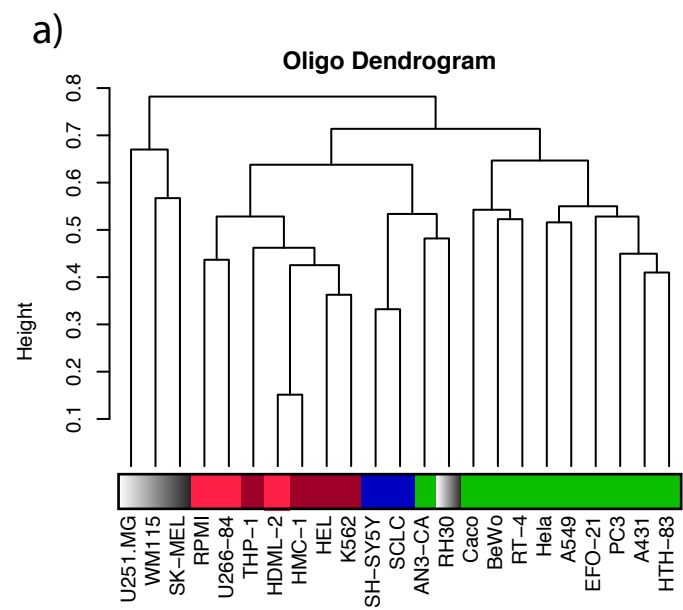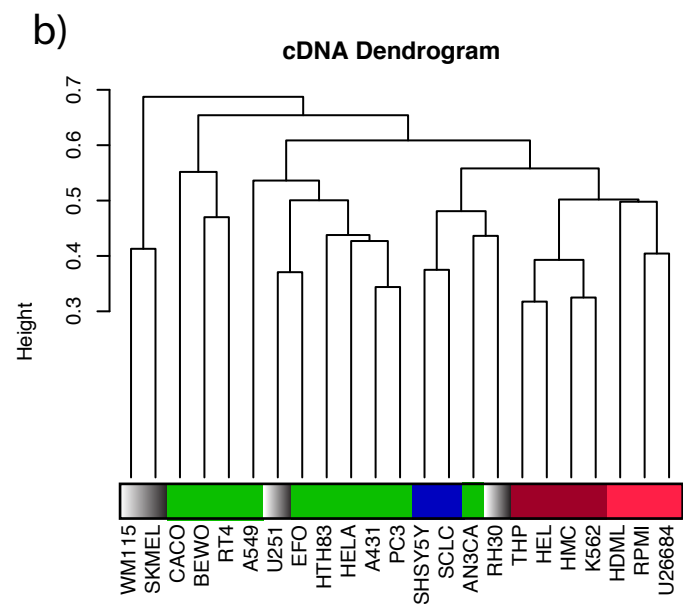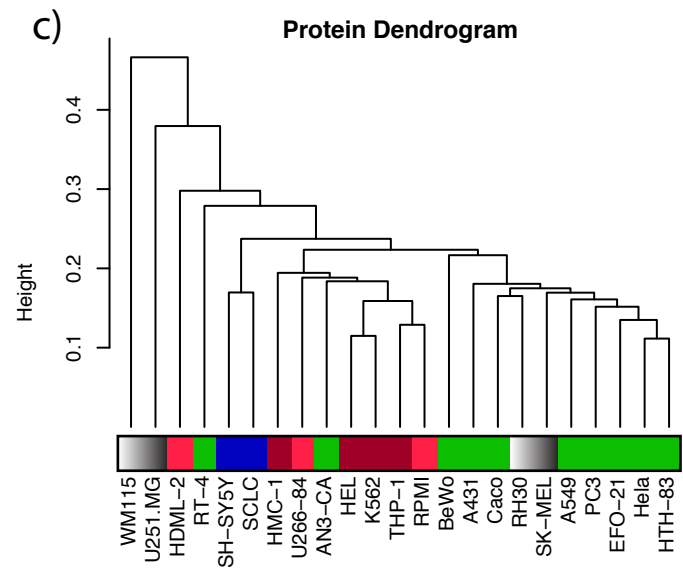

Supplement: Additional file 7 — Dendrograms based on hierarchical clustering of the complete dataset. Dendrograms from hierarchical clustering based on 1066 genes, using the same clustering procedure as for the smaller subset of 167 Ensembl gene IDs. [file 1471-2164-10-365-S7.pdf]

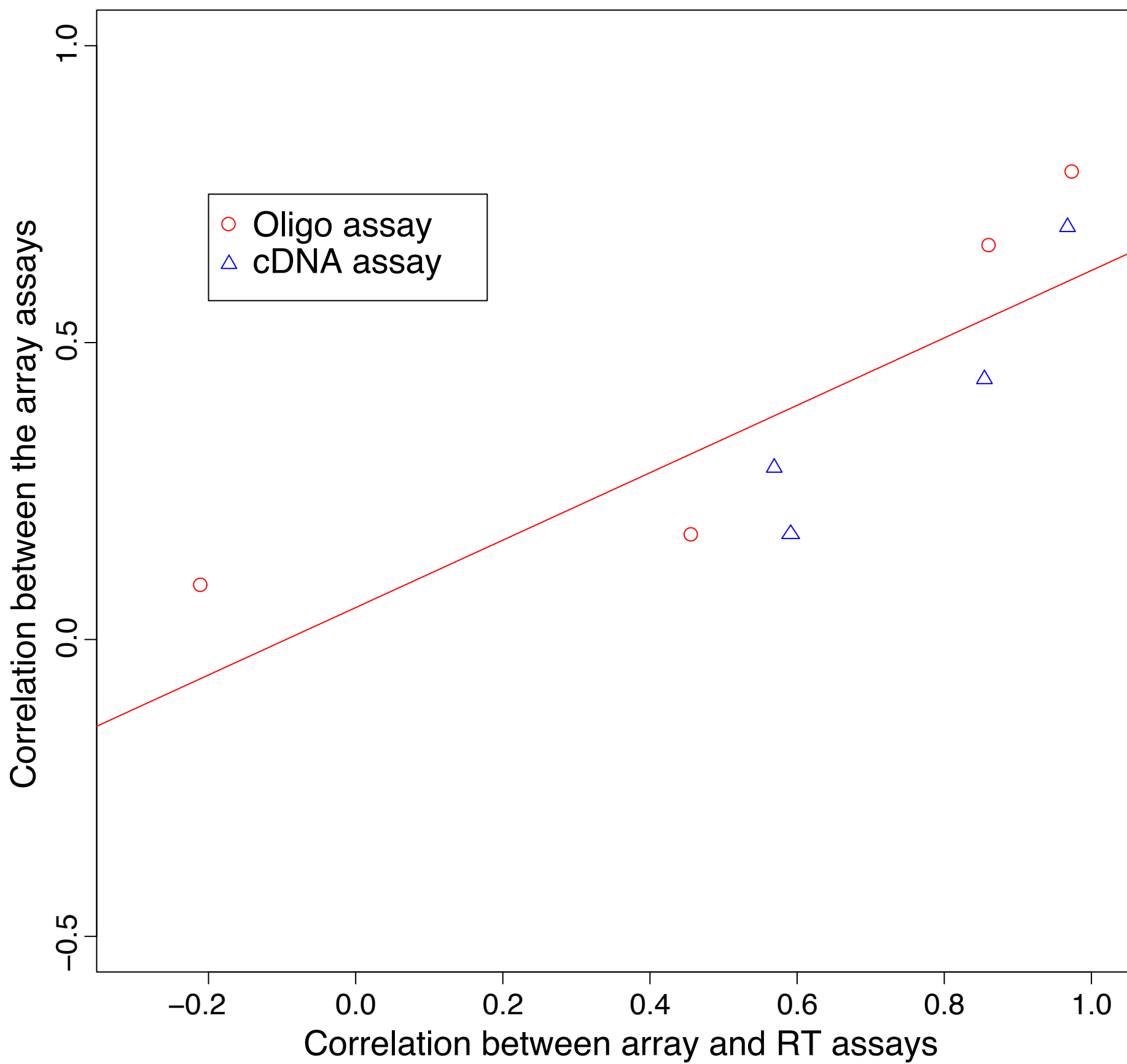

Supplement: Additional file 9 — Linear relationship between correlation coefficients from array and RT-PCR intensity signals. Linear regression of the correlations between the oligo microarray data and the cDNA microarray data (y-axis) and the correlations between each of the array data assays and the RT-PCR assay data (x-axis). The slope is significantly positive (p-value 0.01). [file 1471-2164-10-365-S9.pdf]
